# Xenon Encapsulation in Liposomes, Nanobubbles, and Microbubbles: Delivery Strategies, Preclinical Evidence, and Translational Barriers

**DOI:** 10.3390/pharmaceutics18070855

**Published:** 2026-07-14

**Authors:** Rostislav A. Cherpakov, Vera S. Shashkovskaya, Oleg A. Grebenchikov, Viktoria Sergunova

**Affiliations:** Federal Research and Clinical Center of Intensive Care Medicine and Rehabilitology, V. A. Negovsky Research Institute of General Reanimatology, 107031 Moscow, Russia; verashashkovskaya@googlemail.com (V.S.S.); oleg.grebenchikov@yandex.ru (O.A.G.)

**Keywords:** xenon, microbubbles, liposomes, xenon encapsulation, delivery, preclinical study

## Abstract

Xenon is an active noble gas with anesthetic, neuroprotective and organ-protective properties, but its therapeutic application remains limited by high cost, the need for specialized delivery systems, and the inefficiency of conventional inhalation administration. In this context, Xenon encapsulation into lipid-based nanocarriers, including liposomes and microbubbles, has emerged as a promising strategy to improve targeted delivery, reduce overall Xenon consumption, and enable stimulus-responsive release. This review summarizes current approaches to Xenon loading into liposomes and microbubbles, with emphasis on formulation principles, physicochemical characteristics, stability, acoustic behavior, and ultrasound-triggered release. We further analyze available evidence from preclinical animal models, particularly ischemic stroke, traumatic brain injury, and subarachnoid hemorrhage, where Xenon-loaded carriers have demonstrated the potential to reduce tissue injury and improve functional outcomes. Particular attention is given to the comparative advantages and limitations of liposomal and microbubble-based platforms, including the loading efficiency, circulation behavior, and translational feasibility. Overall, Xenon encapsulation technologies represent a promising direction for the development of localized and potentially more economical Xenon-based therapy. However, further standardization of carrier characterization, dosing strategies, and preclinical protocols is required before these systems can be reliably advanced toward clinical translation.

## 1. Introduction

Xenon is regarded as a potential cytoprotective agent in critical care medicine, as it combines anesthetic activity, a stable hemodynamic profile, rapid pulmonary elimination, and the capacity to attenuate mechanisms of secondary tissue injury [1,2,3].

To date, numerous studies have demonstrated the organ-protective effects of Xenon in systemic and local injuries [4,5,6]. Previously, it was shown that Xenon inhalation is safe and efficient during 24 h of therapeutic hypothermia at 33 °C in comatose patients after successful cardiopulmonary resuscitation. The target end-expiratory concentration was approximately 40%, and no adverse cardiovascular effects were observed [1].

The potential use of Xenon has been considered in several areas of critical care medicine, including therapeutic hypothermia, neurocritical care, neurosurgery, and cardiac surgery [7,8,9,10]. In a preclinical mouse model of traumatic brain injury, inhalation of 75% Xenon with 25% oxygen reduced contusion volume by 43 ± 7% and preserved neuroprotective efficacy when treatment was initiated up to 3 h after injury [7]. In a rat model of subarachnoid hemorrhage, a 50% O_2_/50% Xenon mixture administered 1 h after injury attenuated hippocampal neuronal damage and microglial activation [8]. In a focal ischemia–reperfusion model, inhalation of 0.5 minimum-alveolar-concentration (MAC) Xenon for 60 min reduced ischemic lesion volume by 27% and perifocal edema by 25%, whereas a 120 min exposure did not further enhance the effect [9]. The neuroprotective action was associated with inactivation of glycogen synthase kinase-3β (GSK-3β) and increased levels of its phosphorylated form after 60–120 min of exposure [11], which was accompanied by improved performance in behavioral tests [10].

In clinical studies, Xenon has demonstrated potential neuroprotective and cardioprotective effects. In the randomized Xe-Hypotheca trial involving 110 patients, the combination of Xenon with therapeutic hypothermia reduced white matter injury as assessed by diffusion tensor magnetic resonance imaging (MRI) [2] and was associated with a less pronounced increase in troponin T at 72 h compared with the control group [3]. In patients with severe ischemic stroke, 6 h inhalational sedation with Xenon improved recovery of consciousness, reduced the National Institutes of Health Stroke Scale (NIHSS) score by day 7, and decreased S100B levels by day 3 compared with propofol [12]. In patients with prolonged disorders of consciousness after traumatic brain injury, inhalation of 30 vol% Xenon for 7 days increased the Coma Recovery Scale-Revised (CRS-R) score from 9 to 15 points [13]. In a phase III trial after coronary artery bypass grafting (CABG), Xenon anesthesia was comparable to sevoflurane and was associated with a smaller postoperative increase in troponin I than total intravenous anesthesia [14].

The main limitation of Xenon use in critical care medicine is not its cytoprotective potential, but the technology of delivery. Inhalation provides controllable systemic exposure; however, it requires specialized equipment, sedative–anesthetic gas concentrations, and substantial Xenon consumption. In studies of Xenon anesthesia, gas consumption was particularly high during circuit saturation and maintenance, reaching 9.4 ± 2.1 L at saturation and 18.5–29.6 L during anesthesia maintenance depending on the ventilation mode [15]. Pharmacoeconomic analysis of outpatient Xenon anesthesia also reported a mean consumption of 13 L per procedure and an estimated cost of 196 euros per hour [16]. These limitations are especially relevant for organ protection in critical care, where repeated or prolonged exposure may be required. These limitations have motivated the development of carrier-mediated Xenon delivery systems, including Xenon-containing liposomes and echogenic liposomes [17,18,19,20], Xenon-containing nanobubbles [21], and Xenon-loaded microbubbles, including lipid-shelled and platelet membrane-mimicking microbubble platforms [22,23]. Liposomes and echogenic liposomes are lipid vesicular carriers that can encapsulate Xenon and, in the case of echogenic formulations, support ultrasound-responsive gas release [17,18,19,20]. Xenon-containing nanobubbles and microbubbles represent related but distinct gas-core carrier classes: nanobubbles are submicron carriers with potential advantages for image-guided accumulation and microcirculatory delivery [21], whereas microbubbles are micrometer-scale ultrasound-responsive agents with higher gas-loading capacity and pronounced acoustic sensitivity [22,23]. Ultrasound-triggered release is of particular interest because it provides a potential mechanism for spatially and temporally controlled Xenon delivery near injured tissue, while also requiring further validation of acoustic parameters and safety [18,19,22,23]. The aim of this review is to summarize existing data on Xenon encapsulation into lipid-based carriers, including liposomes, Xenon-containing nanobubbles, and Xenon-containing microbubbles, with emphasis on formulation principles, physicochemical characteristics, acoustic behavior, stimulus-sensitive release, and preclinical evidence of biological efficacy.

**Figure 1:**
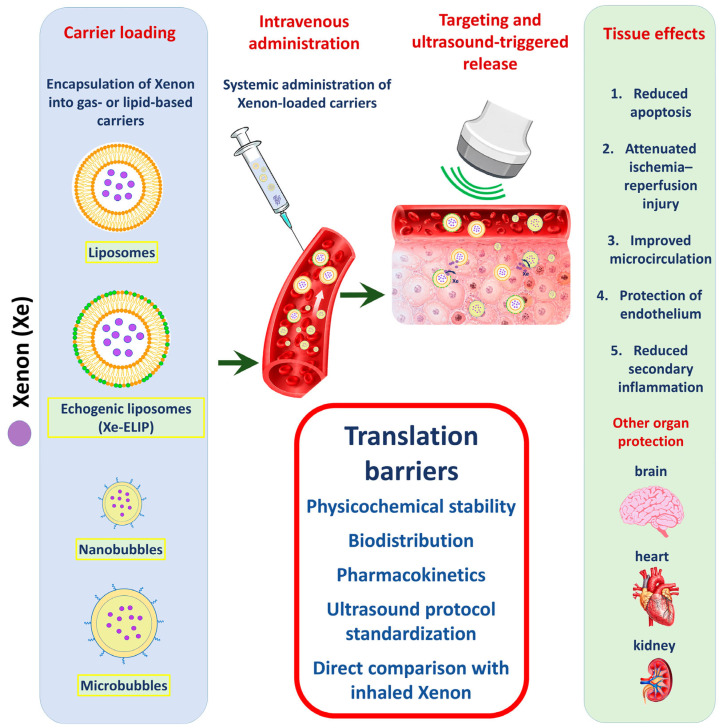
Proposed workflow for carrier-mediated Xenon delivery. Xenon is encapsulated into lipid-based carriers, including liposomes, echogenic liposomes, nanobubbles, and microbubbles. After intravenous administration, Xenon-loaded carriers circulate in the bloodstream and can be exposed to ultrasound to trigger local Xenon release near injured tissue. Expected tissue effects include reduced apoptosis, attenuation of ischemia–reperfusion injury, improved microcirculation, endothelial protection, and reduced secondary inflammation. Key translational barriers include physicochemical stability, biodistribution, pharmacokinetics, ultrasound protocol standardization, and direct comparison with inhaled Xenon.

The general workflow of carrier-mediated Xenon delivery, including carrier loading, intravenous administration, ultrasound-triggered release, expected tissue effects, and key translational barriers, is summarized in Figure 1.

## 2. Targeted Xenon Delivery

The potential of alternative Xenon delivery methods should be assessed, given that, to date, only the inhalation method has been clinically validated. Therefore, alternative Xenon delivery methods are considered promising experimental approaches that require further evaluation before clinical translation. The concept of targeted Xenon delivery in a specialized delivery form pursues two main goals.

First, local delivery can increase the selectivity of Xenon exposure because release occurs in the area of greatest tissue vulnerability, whereas inhalation provides predominantly systemic distribution. Secondly, intravenous administration of Xenon in nanocarriers could improve the technological and clinical feasibility of the approach by reducing the required amount of the active substance [18,24].

A key limitation for producing lipid carriers is their physicochemical stability (particle size, polydispersity index, charge, lamellarity, lipid composition, loading degree, and gas leakage rate). This is crucial for Xenon encapsulation, as Xenon is a small, diffusible, and chemically inert molecule. Therefore, careful attention should be paid to its retention in lipid nanocarriers during storage, loading reproducibility, and tissue distribution [17,18].

Carrier stability should not be interpreted only in terms of particle size. Lipid composition, PEG modification, shell organization, gas solubility, and storage conditions may all influence Xenon retention and leakage from lipid-based carriers. However, the currently available Xenon-loaded liposome, nanobubble, and microbubble studies do not provide systematic quantitative comparisons of Xenon leakage rates across different lipid compositions or PEG-modification strategies. This remains an important formulation-level gap for future studies.

The comparative characteristics of currently described Xenon-containing carrier platforms are summarized in Table 1.

In addition, liposomal carriers can induce immunological responses by activating innate immunity, eliciting cytokine responses, and triggering other immunological effects [25]. As early as 2001, a non-IgE-dependent pseudo-allergic reaction was described with the administration of liposomal preparations [26], and in a number of clinical and experimental studies, undesirable hemodynamic and respiratory effects were noted [27]. A pseudo-allergic reaction associated with activation of the CARPA complement system in therapeutic liposomes has also been previously described, and although short-lived and preventable in individual patients, such reactions can be severe and life-threatening [28]. The encapsulation of Xenon into liposomes serves a fundamental purpose, since Xenon is considered a gas with a favorable tolerability profile, but the carrier can activate the complement system, induce phagocytosis, and increase cytokine expression.

One approach to reducing such risks is optimizing the size of nanocarriers. Several studies have shown that 120 nm nanoparticles persist longer in the bloodstream than 230 nm and 360 nm nanoparticles, a difference attributed to their reduced recognition and clearance by the mononuclear phagocyte system [29]. In the context of developing targeted Xenon delivery systems, reducing the nanocarrier size is a possible approach to increase their efficiency and safety. Jin et al. demonstrated synthesized lipid nanobubbles with an average diameter of 225 ± 11 nm and a Xenon content of 73 ± 2 μL/mL. This carrier format may be promising for local Xenon delivery to areas of blood flow impairment [21].

Another limitation is the controlled gas release from lipid nanocarriers under the influence of external factors, including ultrasound treatment. Ultrasound exposure can cause changes in liposome structure, loss of echogenicity, acoustically induced gas diffusion, fragmentation of the lipid shell, and cavitation effects [30]. This creates the possibility of local Xenon release in the area of interest (the main vessels).

## 3. Delivery Forms of Active Substances

Liposomes are small vesicles that consist of one or more lipid layers and are a promising material for the delivery of hydrophobic and hydrophilic drugs due to their biocompatibility, biodegradability, high bioavailability, and non-toxicity [31,32]. Currently, the Food and Drug Administration (FDA) has approved several liposomal drug delivery systems with broad applications in the treatment of many diseases [33]. Depending on the area of application and lipid composition, the types of liposomes may vary from conventional [34] and long-acting liposomes [35] to modified liposomes with ligands [36]. Liposomes can encapsulate not only hydrophobic and hydrophilic drugs, but also gases, which can be loaded into the liposome core to form liposomal acoustic nanoparticles filled with gas that can be released under the influence of ultrasound [37,38].

The encapsulation of Xenon into liposomes can facilitate targeted delivery of Xenon to tissues, enable controlled gas release under ultrasound stimulation, enhance neuroprotective properties, and reduce the cost of the noble gas. Venkatesh et al. first described the possibility of transporting inert gases within liposomes and used gas-filled liposomes with hyperpolarized ^129^Xe and subsequent MRI [39]. In 2017, a group of scientists led by Klegerman attempted to answer the question of how to measure Xenon in gas-loaded liposomes in the blood [17]. Currently, much attention is paid to the production of echogenic liposomes with encapsulating Xenon (Xe-ELIP), which release gas upon ultrasound exposure [18,19]. Xe-ELIPs were shown to reproducibly contain approximately 19.3 ± 2.8 μL Xe/mg lipid in water and 23.38 ± 7.36 μL/mg lipid in blood. Dandekar et al. synthesized Xe-liposomes with a loading of 15 μL/mg, administered them intravenously to rats in a fixed volume of 0.6 mL, and then released the active component by ultrasound exposure over the common carotid artery [20].

Lipid-shell microbubbles represent an alternative platform for Xenon delivery that is characterized by higher gas capacity and high sensitivity to ultrasound. However, Xenon has a distinct physicochemical limitation: its relatively high solubility can cause it to diffuse out of the gas core of the microbubbles, thereby reducing system stability. For example, co-encapsulation of Xenon with octafluoropropane (OFP), used in the FDA-approved contrast agent Definity, can improve the stability of synthesized microbubbles (shell formed with DSPC and 18:0 PEG2000 PE) containing Xenon [22]. The synthesized Xenon-loaded microbubbles, both with and without the addition of OFP, had a comparable size of less than 10 μm, and co-encapsulation of Xenon with OFP (Xe-OFP-MB) increased the amount of Xenon loaded into the microbubbles (113.1 ± 13.5 μL Xenon/mg lipid for Xe-MB versus 145.6 ± 25.5 μL Xenon/mg lipid for Xe-OFP-MB). The results showed that co-encapsulation of OFP increased particle contrast and stability under undersaturated conditions. Holland et al. noted that Xenon’s high solubility allows it to diffuse out of the resulting microbubbles, whereas co-encapsulation of OFP stabilizes the gas core within the microbubbles. In addition, exposure of the obtained microbubbles to ultrasound at 220 kHz and 6 MHz led to their destruction and gas release [22]. The obtained microbubbles with encapsulated Xenon can be used for further in vivo experiments on neuroprotection in stroke.

Another study focuses on Xenon encapsulation in hybrid platelet membrane-mimicking microbubbles (Xe-Pla-MBs) for targeted delivery to damaged endothelial cells during ischemia–reperfusion injury [23]. Because platelets can bind to damaged endothelium, a platelet membrane coating was used to promote injury-site targeting. Ultrasound was then applied to disrupt Xe-Pla-MBs and release Xenon at the injured renal site. This approach reduced renal fibrosis and improved renal function in ischemia–reperfusion-induced acute kidney injury (AKI) and was associated with decreased markers of cellular senescence, including p53, p16, and β-galactosidase. However, the exact Xenon content of the Xe-Pla-MBs is not specified in the study.

Because the available evidence for Xenon-containing carriers is heterogeneous, it is important to distinguish directly measured outcomes from mechanistic interpretations and effects that have not been demonstrated directly. Table 2 summarizes the main experimental readouts, supported mechanisms, limitations of interpretation, and safe wording for the principal Xenon-containing carrier platforms.

## 4. Key Effects of Targeted Xenon Delivery Based on Preclinical Studies

The analysis of the preclinical data indicates that the evidence supporting different approaches to targeted Xenon delivery is unevenly developed. Xe-ELIPs have been most thoroughly studied in models of cerebral ischemia–reperfusion, subarachnoid hemorrhage, and embolic stroke [18,19,24,40]. At the same time, the Xe-MB and Xe-OFP-MB platforms have engineering advantages, including higher gas load and faster ultrasonic release, and their translational significance is further supported by a large-animal model of TBI [41,42].

Simply categorizing platforms by their effectiveness appears uninformative. To date, there are no direct studies comparing inhalation, liposomal, and microbubble Xenon administration in a single protocol. Therefore, it is advisable to analyze the data using experimental models, taking into account the Xenon formulation, the administration window, and the observed effects.

### 4.1. Cerebral Ischemia–Reperfusion: A Basic Model for Xe-ELIP and Liposomal Development

The most studied model of targeted Xenon application in ischemic injury is transient middle cerebral artery occlusion (MCAO) followed by reperfusion.

Transient MCAO should be distinguished from permanent MCAO because reperfusion introduces additional mechanisms, including oxidative stress, apoptosis, BBB disruption, edema formation, and vascular-barrier injury. In contrast, permanent MCAO is associated with sustained hypoperfusion and progressive infarct expansion. In the carrier-delivered Xenon studies analyzed in this review, permanent MCAO models were not identified; therefore, the available Xe-ELIP and Xe-NB data should be interpreted mainly in the context of transient or reperfusion-associated ischemic injury.

Britton et al. applied this approach using a 2 h MCAO period followed by reperfusion [19]. After reperfusion, the animals were injected with Xe-ELIPs and simultaneously exposed to ultrasound in the zone of the internal carotid artery for 4 min at 1 MHz and 0.18 MPa. The resulting nearly fourfold reduction in normalized infarct volume demonstrates the technical feasibility of this approach and its pronounced biological effect.

A logical continuation was the scientific work by Peng and coauthors, which posed a more clinically oriented question about the optimal time interval and dose of the active substance [24]. Xe-ELIPs were administered at 2, 3, and 5 h after a 2 h MCAO followed by reperfusion at different doses (3.5, 7, and 14 mg/kg) and ultrasound exposure with parameters similar to those of Britton et al. It was shown that, at 2 h, the infarct volume decreased from 16 ± 1.8% in the control group to 2.9 ± 0.7%; at 3 h, to 5.6 ± 1.2%; and at 5 h, to 8.5 ± 1.3%. The injection of Xe-ELIPs at a dose of 14 mg/kg demonstrated the greatest efficacy (Table 3). It was also noted that earlier initiation of therapy was associated with less brain damage. When tested at 3 h after MCAO, a dose of 14 mg/kg showed a stronger neuroprotective effect, and ultrasound exposure led to more effective targeted delivery of Xenon.

In a 2022 study, the injury model was made even more stringent by extending the ischemia time to 6 h and assessing the animals’ condition for 35 days [43]. The primary objective of the study was to determine the efficacy of repeated Xe-ELIP administration following prolonged occlusion. Animals were divided into three groups: sham surgery; stroke with filament removal in 6 h; and stroke with intravenous Xe-ELIP administration during the ischemic phase (at a dose of 6 mg/rat, every hour for 4 h immediately prior to the onset of reperfusion and then once daily from days 1 to 3). Specialized tests were used to evaluate sensorimotor functions, coordination impairments, and anxiety- and depressive-like behavior over time. Additionally, on day 35, a morphological study of 2,3,5-triphenyltetrazolium chloride (TTC)-stained brain sections was performed along with an assessment of the cytokine profile and microbiota status.

Neurological and cognitive impairments were assessed at the early period, as simple sensorimotor tests often show maximum sensitivity at that time [44]. At later stages, an assessment was performed with an emphasis on anxious–depressive behavior as a characteristic indicator of the post-stroke phenotype [45].

When assessing sensorimotor asymmetry in the context of stroke, repeated administration of Xe-ELIPs significantly reduced sticker removal time: without therapy, it was 50.33 ± 15.09 s, and after repeated administration of Xe-ELIPs, it was 23.84 ± 10.07 s (*p* < 0.016). In the coordination test, Xenon use also had a beneficial effect: on the 3rd day, the retention time was 98.22 ± 6.61% of the baseline level, compared with 63.09 ± 7.86% in the stroke group (*p* < 0.01). It was shown that on the 28th day after a 6 h MCAO, the animals demonstrated a decrease in the proportion of time spent in and the number of entries into the open arms of the elevated plus maze, consistent with anxiety-like behavior. Repeated administration of Xenon liposomes increased both parameters, while the number of entries into the closed arms did not change significantly, reducing the likelihood of a simple motor interpretation of the effect. On day 35 of the forced swim test, active swimming time decreased in untreated animals, whereas in the Xe-ELIP group, it increased approximately twofold compared with the stroke group (Table 4).

During the morphological study, the authors also noted that no significant morphological damage was observed in TTC sections after Xenon treatment, whereas in animals with stroke, a tissue scar was visible in the area of the presumed penumbra and ischemic core. The cytokine profile at 35 days did not show consistent, statistically significant differences. Separately, the authors noted a numerical increase in IL-6 in the Xe-ELIP group, which may be associated with changes in the intestinal microbiota induced by Xenon liposomes. It was shown that in the acute cerebrovascular accident (MCAO) group, IL-4 and IL-13 levels decreased by half, while IL-1β and IL-10 levels did not differ significantly from reference values, and the concentration of Monocyte Chemoattractant Protein-1 (MCP-1) increased 1.5-fold. In the MCAO group treated with Xe-ELIPs, IL-1β levels remained unchanged compared to the control group while IL-4, IL-13, and IL-10 levels were restored relative to the control group. The most pronounced increase was observed for IL-6 (almost fivefold compared to the control group).

However, all of the above models reproduced ischemia with controlled restoration of blood flow; for clinical translation, it is important to test the combination of targeted Xenon and drug-induced reperfusion. For this purpose, embolization of the middle cerebral artery (MCA) lumen with a pre-prepared blood clot was performed, followed by alteplase administration [40]. The work was divided into three parts: to determine whether a lipid carrier can influence the effectiveness of thrombolysis and whether there is synergy between targeted delivery of Xenon and thrombolysis in the early (2 h after the thrombosis) and late (4 h after the thrombosis) stages of ischemia.

In the first stage, blood clots weighing 120 ± 3.3 mg were prepared in vitro from 0.5 mL of Yucatan minipig blood by incubation for 3 h at 37 °C. To determine the effects of liposomes, the clots were transferred to 1 mL of human plasma supplemented with human plasminogen (1 U/mL) and treated with either alteplase (80 μg/mL) or alteplase combined with Xe-ELIPs (23.38 ± 7.36 μL Xe/mg lipid, 6.4 mg lipid per rat). The clots were weighed 30 min after incubation in a water bath at 37 °C, and mass loss was recorded. The addition of Xenon did not affect the mass loss: in the control group, it was 34 ± 3.0%, and in the group with Xe-ELIPs, it was 33 ± 8.8%, which allowed us to proceed to the next stage.

MCA embolization was induced under Doppler control by intraluminal injection of a rat arterial clot fragment (12 × 0.35 mm) prepared from 200 μL of blood with thrombin and CaCl_2_ with treatment initiated 2 or 4 h after confirmation of occlusion (Table 5).

For therapy initiated 2 h after occlusion, the normalized lesion volume measured on day 3 was 16.5 ± 6.0% in untreated animals, 5.6 ± 3.0% after alteplase alone, and 2.4 ± 0.9% after combined alteplase–Xenon treatment, with significant differences relative to both the untreated and alteplase-only groups. The motor deficiency also clearly regressed in both groups against the background of standard therapy and its combination with Xenon.

With delayed initiation of specific therapy, late thrombolysis led to a sharp increase in hemorrhagic complications: in the untreated group, their incidence was 13% versus 58% in the alteplase group. The results obtained for this type of complication partially represented a logical extension of an earlier study in which Xenon inhalation in the post-reperfusion period also reduced the incidence of hemorrhagic complications [46]. At the same time, the combination of thrombolytic therapy and Xenon reduced the incidence of hemorrhages to 20%.

The normalized volume of the formed lesion in the control group was 20.7 ± 6.1%, even with the late use of alteplase (7.6 ± 2.7%), but in combination with Xenon, the volume of necrosis was virtually no different from early reperfusion and was 2.0 ± 1.4% (which was significantly lower compared to the other two groups). In the peri-infarct zone, the number of TUNEL-positive cells was 593 ± 46 cells/mm^2^ in untreated animals, 324 ± 37 cells/mm^2^ in the alteplase-only group, and 155 ± 81 cells/mm^2^ in the alteplase–Xenon group, suggesting a marked reduction in cell death with combined therapy.

The authors simultaneously assessed the expression of matrix metalloproteinase 9 (MMP9) in the cerebral microvascular wall. With alteplase alone, MMP9 immunoreactivity was prominent in the cerebral microvascular zone and coincided with the α-actin-positive contour of the vascular wall. The addition of Xenon liposomes was accompanied by a significant reduction in this signal, which was associated with a lower incidence of hemorrhagic complications and could indicate protection of the vascular-barrier component of ischemic injury.

The preclinical relevance of Xenon as a targeted cytoprotective agent in hemorrhagic cerebrovascular injury is also demonstrated by Miao et al., who compared the effectiveness of Xe-ELIPs (20 μL Xe/mg lipid, 6 mg per animal) with empty nanocarriers and Xenon-saturated solution in a subarachnoid hemorrhage model. Therapeutic efficacy was assessed by measuring hemorrhage volume and quantifying TUNEL-positive cells. Empty ELIPs and Xenon-saturated solution did not produce a significant therapeutic effect, whereas Xe-ELIPs reduced hemorrhagic volume from 327 ± 25 to 165 ± 9 μL, and decreased the number of TUNEL-positive cells from 396 ± 22 to 72 ± 9 compared with the untreated subarachnoid hemorrhage group [18].

Xenon-containing lipid nanobubbles have emerged as a separate strategy for targeted Xenon delivery in ischemic stroke. Jin et al. synthesized particles with an average diameter of 225 ± 11 nm, a concentration of approximately 1.95 × 10^9^ particles/mL, and a Xenon content of 73 ± 2 μL/mL. In a mouse ischemia–reperfusion model, 1 h MCAO was followed by intravenous administration of phosphate-buffered saline (PBS), Xenon-saturated saline, or Xenon-containing nanobubbles after recanalization. Nanobubbles accumulated in the ischemic injury zone, provided ultrasound visualization of the lesion, and improved microcirculatory recovery: by the 270th min, blood flow in the lesion zone reached 88 ± 2% of normal levels, compared with 77 ± 2% with the Xenon-saturated solution and 75 ± 3% in the control group. This was accompanied by a decrease in infarct volume, a reduction in the severity of apoptosis, and an improvement in neurological status [21].

Taken together, these studies show that Xenon-containing carriers have been evaluated in heterogeneous cerebrovascular injury models, including transient MCAO with reperfusion, prolonged ischemia–reperfusion, embolic MCAO with pharmacological reperfusion, subarachnoid hemorrhage, and mouse MCAO treated with Xenon-containing nanobubbles. Because these models differ in injury mechanism, therapeutic window, carrier type, and measured outcomes, the available evidence should not be interpreted as a single uniform dataset. The main preclinical studies, experimentally measured outcomes, mechanistic support, and interpretation used in this review are summarized in Table 6.

In parallel, potential strategies for targeted Xenon delivery in TBI were explored with particular emphasis on the microbubble platform, as ultrasound-triggered Xenon release at the carotid artery level was well suited for early delivery to the injured brain region.

### 4.2. The Use of Xenon in the Model of TBI

The first work in this direction was the study by Coburn et al., which used organotypic hippocampal slices with focal mechanical injury in the CA1 region [47]. The authors analyzed the total damage and secondary component separately, excluding the immediate impact zone from the calculations. It was demonstrated that a gas mixture containing 75% Xenon reduced damage even when exposure began 3 h after injury, with the primary effect being associated with a reduction in delayed cell death beyond the primary zone of mechanical injury. The effects of Xenon were studied at the whole-body level by Campos-Pires et al., who included several experimental blocks (Table 7) [7].

These blocks were performed in a controlled cortical contusion model in male C57BL/6N mice and included assessments of the therapeutic window, concentration dependence, early neurological deficit, contusion volume, and long-term motor function. Early neurological deficit was assessed using a 15-point neurological scale that characterizes locomotor and vestibulomotor function. To quantitatively assess the extent of structural brain damage 24 h after injury, the morphology of the brain lesion was examined, followed by the calculation of the contusion volume. Physiological safety during exposure was monitored using blood pressure, heart rate, and body temperature. Early recovery dynamics were analyzed during days 1–5 after injury using repeated neurological assessments. The CatWalk-XT system was used to characterize motor coordination, movement speed, and gait parameters in animals and to assess long-term motor function at 1 month.

Each experimental block tested a distinct aspect of Xenon’s effect and consistently refined the results of the previous phase. In the first block, the Xenon inhalation began before injury and continued afterward. This design simulated conditions in which Xenon was already present in the body at the time of injury, allowing for the assessment of the maximum potential protective effect. Neurological outcome in 24 h improved by 40 ± 11% and contusion volume decreased by 43 ± 7% compared to the control group.

To more closely approximate realistic clinical scenarios, Xenon was administered only after TBI. Inhalation of 75% Xenon/25% O_2_ was initiated 15 min after the injury and continued for 3 h. In this scenario, the neurological outcome at 24 h improved by 36 ± 6%, and contusion volume decreased by 19 ± 7% compared to the control group.

The next stage of the study aimed to define the therapeutic window for Xenon after TBI by delaying inhalation onset by 15 min, 1 h, 3 h, or 6 h after injury. Improvement in neurological outcome was maintained at 15 min and 1 h post-injury, reaching 33% and 46%, respectively. With delays of 3 and 6 h, the effect persisted only as numerical differences. Neurological outcomes improved by 27% and 26%, respectively, but did not reach statistical significance. However, the morphological effect was more stable with contusion volume reduced by approximately 20% when therapy was initiated at 15 min, 1 h, and 3 h, whereas with a delay of up to 6 h, the effect virtually disappeared.

Given the high cost of Xenon, assessing the effective concentration range was particularly important. An analysis of the concentration dependence showed that reducing the volume of the contusion did not require use of the maximum gas concentration.

When therapy was initiated 15 min after TBI and lasted for 3 h, lesion volume decreased by 19 ± 1% with 30% Xenon, by 17 ± 1% with 50% Xenon and by 19 ± 2% with 75% Xenon. Neurological outcome significantly improved with 30% and 75% Xenon, while only a trend was observed with 50%.

To assess the duration of the effect, the neurological outcomes were analyzed at 5 days and 1 month after injury. The improvement persisted for up to 4 days, while differences with controls were no longer statistically significant on day 5.

This result is consistent with behavioral assessments in rodents, as severe neurological deficits are more readily detected early after injury, whereas the sensitivity of simple scales gradually decreases as spontaneous recovery progresses.

Long-term outcomes were assessed over 1 month after TBI. Rotarod revealed no differences between the groups, while a more sensitive gait analysis revealed persistent impairments after TBI. Movement speed decreased to 43 ± 3 cm/s after TBI compared with 53 ± 3 cm/s in sham-operated animals, whereas Xenon-treated animals demonstrated preserved movement speed of 50 ± 2 cm/s, not significantly different from sham values. TBI also reduced limb swing speed, especially for the right hind paw, by 22 ± 2% (*p* < 0.01), while this deficit was not significant after Xenon treatment.

A detailed examination of this work is warranted because it was not limited to demonstrating the protective effect of Xenon and enabled evaluation of therapeutic window, concentration dependence, and the early and late effects of exposure to mechanical brain injury.

The same group subsequently expanded the experimental approach by evaluating the effects of Xenon in an in vitro model of blast-induced TBI. Organotypic hippocampal slices from C57BL/6N mice were subjected to a single Friedlander-profile shock wave. In the neuroprotective experiment, the pressure was 55 kPa, which caused a reproducible increase in damage over 72 h. An inhalation mixture containing 50% Xenon was administered 1 h after blast exposure, reducing the severity of damage by 47 ± 12% at 24 h, 31 ± 7% at 48 h and 39 ± 7% at 72 h. Moreover, the zone of damage according to propidium iodide coincided with the activation of caspase-3, indicating the involvement of the apoptotic component of cell death [48].

It should be emphasized that the available TBI literature is not evenly distributed across carrier systems. Small-animal brain contusion and blast-type injury studies mainly support the biological neuroprotective potential of Xenon itself, whereas direct targeted-carrier data in TBI are currently represented primarily by microbubble-based large-animal studies. In contrast, Xe-ELIP evidence is better developed in cerebrovascular ischemia–reperfusion, embolic stroke, and subarachnoid hemorrhage models, but direct in vivo Xe-ELIP data in brain contusion or blast-type TBI models remain limited. Therefore, liposomal and microbubble-based carriers should not be interpreted as equivalent in the TBI setting. Liposomal carriers may offer advantages related to nanoscale circulation and potentially longer intravascular persistence, whereas microbubbles provide higher gas-loading capacity and stronger ultrasound responsiveness but require careful control of acoustic exposure, vascular safety, and cavitation effects. At present, the absence of direct head-to-head comparisons between Xe-ELIPs and Xe-MBs in TBI prevents a definitive conclusion regarding the superior carrier platform for cranial injury.

The first study of targeted Xenon delivery was conducted in a pilot study by Hwang et al. in piglets, which is of great interest both as the first study of a targeted Xenon delivery system in the form of microbubbles and as a large-animal model [41]. The injury model used was a controlled cortical impact on the rostral gyrus after right-sided craniectomy, resulting in focal mechanical damage.

Microbubbles (DBPC + DSPE-PEG5000, 9:1 molar ratio) were used as the targeted delivery component. The Xenon content in the suspension was 4.5 μmol/mL, resulting in an estimated concentration of approximately 10^8^ microbubbles/mL, with perfluorobutane used as a control. Microbubbles were intravenously injected at 1, 3, and 24 h after TBI. At each stage, an 8 min infusion was performed, and the microbubble concentration in both groups was adjusted to approximately 10^8^–2 × 10^8^ particles/mL. Following intravenous injection, a portable Lumify Philips ultrasound probe was applied to the carotid artery, serving as a trigger for gas release.

The effectiveness of the method was assessed using MRI scans of the brain to measure the volume of the formed nucleus and perifocal edema on days 1 and 5, and by morphological examination of the injured area on day 5, assessing endothelial proliferation and perivascular inflammation. Xenon administration resulted in a limited subacute enlargement of the traumatic lesion and was accompanied by a reduction in reactive vascular changes. Perivascular inflammation was also reduced but did not reach statistical significance (Table 8).

Given the small sample size, the study did not address questions of dosing, pharmacokinetics, and long-term functional outcome. Nevertheless, the possibility of targeted Xenon delivery to the target organ in the form of microbubbles, the measurable effects on MRI and morphology, and the significantly lower absolute gas consumption compared to inhalation regimens represent an important step in further scientific research.

Another key study was by Shin et al., in which the authors aimed not only to substantiate the effectiveness and delivery methods but also to determine which links in the pathogenesis after TBI are affected by Xenon [42]. Essentially, the study addressed all levels of damage assessment after TBI—from cellular to macroscopic brain tissue assessment using MRI and morphological data. The study itself was divided into two parts: an in vitro study using BEND3 endothelial cells and an in vivo study using 4-week-old piglets.

In vitro, it was tested whether Xenon microbubbles could limit glutamate-induced disruption of endothelial tight junctions, which is important for the vascular-barrier component of TBI. Cells were treated with 80 mM glutamate for 24 h, then supplemented with control or Xenon microbubbles at 20% of the total culture medium volume for an additional 24 h. Zonula Occludens-1 (ZO-1) was then stained and its expression assessed by confocal microscopy. It was shown that exposure to 80 mM glutamate resulted in a significant decrease in ZO-1 levels, almost twofold compared to the control. Xenon treatment maintained ZO-1 expression at levels similar to those observed in control cells, indicating partial preservation of endothelial tight junction integrity under pronounced glutamate-induced excitotoxicity. By contrast, perfluorobutane control microbubbles failed to restore ZO-1 expression compared with exposure to 80 mM glutamate alone.

For in vivo experiments, the authors replicated the design of Hwang et al.’s study, significantly expanding the methods used to assess damage and the effects of Xenon. Controlled cortical impact was also used to model TBI in piglets, and the administration regimens and microbubble compositions were similar to those used previously [41]. Stimulus-sensitive delivery was achieved by applying ultrasound at the level of the carotid artery, which resulted in Xenon release immediately prior to brain entry. Parameters measured included early cerebral edema and its dynamics, overall tissue diffusion, the vascular component of inflammation, and astrocytic and microglial reactivity (Table 9).

Thus, the study by Shin et al. demonstrates that the effect of Xenon microbubbles in large-animal TBI was most consistently evident in the vascular-barrier block. According to diffusion MRI data, this was expressed primarily as an early reduction in signs of vasogenic edema and, according to morphology, as a reduction in reactive vascular changes, perivascular inflammation, astrocytic reactivity, and fibrinogen extravasation. The absence of significant changes in Iba1 and axial diffusivity makes this conclusion more precise: the study primarily supports a vascular-endothelial rather than a universal anti-inflammatory interpretation of the effect.

In conclusion, Xenon research (both inhalation and targeted delivery) has focused primarily on the brain. However, other studies focus on the effects of Xenon in microcarrier form on extracranial injuries [23,49].

## 5. Targeted Xenon Delivery in Models of Renal and Myocardial Injury

Despite the predominance of neurovascular models, targeted Xenon delivery is not limited to brain injury. Extracranial data are currently represented by fewer studies, but they are important for assessing the universality of the cytoprotective concept. In this area, models of ischemia–reperfusion organ injury are of particular interest, as they capture the interplay among endothelial dysfunction, local inflammation, oxidative stress, and secondary tissue remodeling.

For example, Yang et al. used a renal ischemia–reperfusion model to test the hypothesis that targeted Xenon delivery to the damaged endothelium could limit acute renal tissue injury and subsequent profibrotic remodeling [23]. In 1 h after left kidney ischemia and subsequent reperfusion, 400 μL of Xe-Pla-MBs were administered intravenously, and gas release was stimulated with ultrasound for 10 min. The carrier bound to the damaged endothelium via its platelet membrane, and a therapeutic effect was observed only with the combination of Xenon, platelet membrane, and ultrasound disruption. This was accompanied by decreases in the pathological score, creatinine, urea, and the cellular senescence markers p53/p16/β-galactosidase (β-gal), whereas Xenon microbubbles without a platelet membrane, empty platelet microbubbles, and a Xenon-saturated solution had no significant effect. Creatinine and urea levels were reduced by almost half after intravenous administration of Xe-Pla-MBs compared with the ischemia–reperfusion group, indicating reduced azotemia and partial recovery of kidney function. Reduced microbubble adhesion to damaged endothelium and less severe endothelial damage were also noted during repeated ultrasound exposure after intravenous administration of Xe-Pla-MBs in the ischemia–reperfusion model.

Validation of the model included not only creatinine and urea, but also the tubular damage markers Neutrophil Gelatinase-Associated Lipocalin (NGAL) and Kidney Injury Molecule-1 (KIM-1), which increased after ischemia–reperfusion. Separately, the authors confirmed the endothelial nature of the damage: after ischemia–reperfusion, syndecan-1 and hyaluronic acid levels increased, and fibrinogen and collagen IV signals were enhanced in kidney tissue [23].

This study demonstrated that targeting can be achieved not only by an external ultrasound trigger but also by biological modification of the carrier itself. The platelet-mimetic shell enhanced the fixation of microbubbles at the site of endothelial damage, and the therapeutic effect was evident only with a combination of three components: Xenon, platelet targeting, and ultrasonic destruction of the carrier.

The cardioprotective branch of targeted Xenon delivery is currently represented only by limited preliminary evidence. In a conference abstract by Yin et al., Xe-ELIPs were evaluated in a rat model of acute myocardial infarction by ligation of the anterior descending coronary artery [50]. Xenon-containing liposomes were injected intravenously 45 min after infarction, immediately following reperfusion. The reported gas load was 190 μL Xe/mg lipid. Efficacy was assessed by echocardiography at baseline, after the simulated infarction, and on day 7, as well as by infarct volume measurement by TTC staining. The authors reported that Xe-ELIPs reduced left ventricular end-systolic volume, increased cardiac output, and decreased infarct volume. However, because these findings were published only as a conference abstract and the available numerical dataset is incomplete, including limited information on injection volume, lipid dose, pharmacokinetics, and group-level outcome data, this evidence should be regarded as preliminary and hypothesis-generating. It should not be weighted equally with full peer-reviewed experimental studies.

Thus, extracranial targeted Xenon delivery remains supported by limited and heterogeneous evidence and is substantially less developed than neurovascular models. The renal Xe-Pla-MB study currently provides the most informative extracranial model, combining ultrasound-triggered Xenon release with platelet membrane-mediated adhesion to damaged endothelium. By contrast, myocardial Xe-ELIP evidence remains preliminary because it is based on abstract-only data and lacks complete experimental reporting. These platforms also differ mechanistically: Xe-Pla-MBs use biological endothelial targeting via the platelet-mimetic shell, whereas Xe-ELIPs mainly rely on intravascular administration and ultrasound-triggered release. Therefore, platelet-mimicking microbubbles and echogenic liposomes should be considered complementary, but not directly equivalent, extracranial Xenon delivery systems. Further studies are needed to compare their biodistribution, gas retention, ultrasound release efficiency, organ-specific safety, and therapeutic efficacy. Direct comparisons of intravenous carriers with inhaled Xenon in extracranial models are not yet available; therefore, the current value of these studies lies in expanding the range of possible organ-protection targets rather than in demonstrating clinical or translational equivalence.

## 6. Safety and Translational Barriers of Xenon-Loaded Carriers

In this review, we focused on Xenon encapsulation into different carrier systems, including liposomes, nanobubbles, and microbubbles, with emphasis on their physicochemical characteristics, acoustic behavior, stability, and preclinical efficacy. However, the clinical translation of Xenon-loaded carriers also requires a separate analysis of safety limitations. These limitations are related not only to Xenon itself, which is chemically inert, but also to carrier composition, surface biology, complement activation, coagulation, biodistribution, repeat-dose exposure, and ultrasound-triggered cavitation.

For example, the Xe-Pla-MBs combine injury-site targeting followed by ultrasound-triggered Xenon release; however, the administration of Xe-Pla-MBs may trigger adverse reactions that are not associated with Xenon itself as it is an inert gas, but with the platelet membrane, adhesion receptors, and interactions involving the endothelium, the complement system, coagulation and ultrasonic cavitation. Xe-Pla-MBs may potentially have a prothrombotic risk due to mimicking the ability of the platelets to recognize the damaged endothelium [23]. The platelet membrane not only contains adhesive receptors involved in binding to fibrinogen, collagen, and the damaged endothelium, but also carries P-selectin, integrin, and receptor complexes which can mediate immune evasion and vascular adhesion [51]. Another concern of the Xe-Pla-MBs is the possible formation of a procoagulant surface due to the externalization of the phosphatidylserine on the outer leaflet of the platelet membrane.

Phosphatidylserine participates in the activation of thrombin and subsequent fibrin formation [52]. Therefore, if the synthesis of Xe-Pla-MBs or Xe-ELIPs induces phosphatidylserine exposure, the Xe-Pla-MBs may acquire the platelet-like procoagulant phenotype rather than acting only as passive injury-site carriers and require strict control of “background” activity in the bloodstream [53].

The immune reactions may be associated with the microbubble shell, PEG/lipids, residual platelet proteins, the protein corona and complement. Xenon itself, being a noble gas, is likely not an antigen; however, the Xe-Pla-MB and the Xe-ELIP platforms are not immunologically neutral by definition. In targeted lipid microbubbles, C3/C3b is a major serum protein associated with the microbubble surface, and C3/C3b deposition depends on the physicochemical architecture of the carrier (microbubble size, PEG-brush organization). It was demonstrated that C3/C3b may be the primary serum factor binding to the surface of the microbubbles and this can lead to pseudo-allergic reactions, accelerated clearance and phagocytosis [54]. Platelet membranes can inhibit phagocytosis; however, in the case of incomplete coverage, altered protein orientation, or complement fixation, the microbubbles can be rapidly taken up by macrophages in the spleen and liver. Therefore, preservation of the membrane proteins, correct receptor orientation, and complete membrane coverage are critical for platelet membrane-coated systems [51]. P-selectin exposed on the platelet membrane can interact with P-selectin glycoprotein ligand 1 (PSGL1) which is expressed on leukocytes, including neutrophils and monocytes. This interaction can enhance the formation of platelet–leukocyte aggregates and can amplify thromboinflammatory signaling by promoting neutrophil recruitment and monocyte and endothelial activation [55,56].

The ultrasound-triggered release of Xenon and subsequent destruction of the microbubbles can trigger local mechanical effects, such as cavitation, microstreaming, sonoporation, and increased vascular permeability. For ultrasound-responsive xenon carrier platforms, the balance between Xenon release and endothelial safety depends on acoustic pressure or mechanical index, frequency, pulse duration, duty cycle, exposure time, microbubble concentration, shell composition and vascular status of the target tissue [22,57]. Previously it was demonstrated that excessive acoustic energy can exacerbate endothelial damage, hemorrhage, thrombosis and secondarily initiate coagulation [57,58]. This is particularly crucial for the central nervous system as the ultrasound stimulation with the administration of the microbubbles can alter the permeability of the blood–brain barrier (BBB). While this can be beneficial for the delivery, it necessitates the control of the acoustic parameters and microbubble dosage, as well as monitoring for microhemorrhage or edema [59]. The same agent may be safe under mild exposure conditions and damaging under conditions that induce intense inertial cavitation. Therefore, Xe-MBs are not a medical product but a device–drug combination requiring the validation of not only the formulation but also the ultrasound system, the exposure parameters and the cavitation [60]. Consequently, future translational studies should report Xenon release kinetics under graded acoustic parameters together with dose-dependent vascular safety endpoints, including endothelial viability, permeability, hemolysis, platelet/coagulation activation, microhemorrhage, fibrin deposition and histological evidence of microvascular injury. Therefore, the therapeutic effect of ultrasound-triggered Xenon release and the risk of vascular injury are not independent phenomena but represent two sides of the same acoustic bioeffect continuum.

Thus, the main barriers to clinical translation are not limited to biological efficacy. They include reproducible Xenon loading, gas retention during storage, particle size control, shelf life, prevention of off-target release, and validation of ultrasound parameters for safe and efficient Xenon release. Since Xenon-loaded carriers, particularly Xe-ELIPs, Xe-MBs, and Xe-Pla-MBs, function as drug–device combination systems, clinical translation will require integrated evaluation of carrier formulation, Xenon dose, acoustic parameters, cavitation monitoring, complement activation, thrombogenicity, biodistribution, pharmacokinetics, repeat-dose safety, and organ-specific toxicity.

Addressing these barriers will be important for determining whether targeted Xenon delivery can progress from preclinical feasibility toward clinical translation.

## 7. Clinical Limitations and Translational Barriers of Xenon-Loaded Carrier Systems

The application of Xenon-loaded systems, including Xe-ELIPs, Xe-MBs, and Xe-Pla-MBs, in clinical practice appears promising but currently remains at the preclinical stage. These limitations are related not only to the biological effects of Xenon and the carrier platforms, which were discussed earlier in this review, but also to the delivery methods, the safety of microbubbles and liposomes, the ultrasound parameters required for triggered gas release, and the potential regulatory classification of these systems as drug–device combination products. Regarding Xe-MBs, Xe-ELIPs and Xe-Pla-MBs, the preclinical data is demonstrated; however, for Xe-MBs, the authors themselves outline a need for larger randomized preclinical studies, as well as Xenon quantification in blood and brain, pharmacokinetics, dosing regimens, and biosafety assessment [22]. The clinical limitation of Xe-ELIPs is focused on unresolved issues regarding clinical dosage, repeated administration, and safety in patients with acute vascular injury and scalable manufacturing [18]. A clear clinical limitation of Xe-Pla-MBs is the necessity of proof regarding the absence of thrombogenicity, off-target adhesion, and immune–thrombotic reaction.

Unlike conventional pharmaceutical agents, Xenon is a gas; therefore, its clinical translation requires characterization not only of the injected volume of microbubbles or liposomes, but also of the actual amount of Xenon loaded into the carrier, the fraction released after ultrasound exposure, the concentrations achieved in blood, tissues, and target organs, and the fraction retained during storage. Shekhar et al. demonstrated that co-loading of Xenon with OFP improves the stability and acoustic properties of Xe-OFP-MBs; however, without standardized quantification of the gas payload and leakage rate, it is impossible to reliably dose the therapy [22].

All three platforms (Xe-MBs, Xe-ELIPs, Xe-Pla-MBs) require acoustic activation or visualization. For ultrasound-triggered release of Xe from Xe-MBs, the ultrasound was applied at the level of the carotid artery to release Xenon into the cerebral circulation [41]. However, clinical implementation requires standardization of frequency, mechanical index, acoustic pressure, focal depth, vessel localization and cavitation monitoring. Thus, ultrasound parameters may simultaneously determine both Xenon release efficiency and vascular safety. To improve cross-study readability, the ultrasound-triggering parameters reported in the available Xenon-loaded carrier studies are summarized in Supplementary Table S1. When Xenon platforms are applied to the brain, the safety of the vascular wall and BBB becomes the critical limitation. Potential adverse effects associated with focused ultrasound-induced BBB opening by microbubbles have been previously demonstrated, including microhemorrhage, ischemia resulting from vasoconstriction, and direct cellular damage from thermal or mechanical forces [61]. This does not indicate that Xe-MBs will cause such limitations; however, clinical implementation requires a safe acoustic range for each specific indication—TBI and stroke. This will be challenging particularly in patients with pre-existing endothelial damage, BBB disruption, coagulopathy, thrombocytopenia, or systemic inflammation.

Another clinical limitation of the use of Xe-Pla-MBs is the hemostatic constraints. For standard Xe-MBs, the primary hemostatic concerns involve the microbubble size, concentration, aggregation, endothelial contact, and cavitation. For Xe-Pla-MBs, the constraints are significantly stricter due to the adherence of platelet membrane to the damaged endothelium. This creates clinical risks such as off-target adhesion in the inflamed vessels, interactions with collagen and fibrinogen, platelet–leukocyte aggregation, local thrombin generation and microvascular thrombosis [62,63]. Yang et al. used the platelet mimicry to facilitate delivery to sites of renal ischemia–reperfusion injury; however, this mechanism needs extensive clinical hemocompatibility assessment prior to application in patients with sepsis [23].

## 8. Conclusions

Current research on Xenon is gradually shifting from its exclusive role as an inhalation anesthetic to a broader concept of a cytoprotective agent. Clinical and preclinical data already support its effects in the context of limiting excitotoxicity, ischemia–reperfusion injury, apoptosis, neuroinflammation, and vascular endothelial dysfunction. However, the delivery method remains a key limitation to scalability: traditional inhalation requires specialized equipment and significant gas consumption, and creates a systemic rather than targeted field of action.

In this context, liposomal, nano-, and microbubble forms of Xenon are a logical development of the already established cytoprotective concept. Preclinical models have shown that Xenon can be packaged in lipid carriers, released by ultrasound, and retain its biological activity after intravenous administration. The most mature line of evidence to date relates to cerebral ischemia–reperfusion and other neurovascular models, in which Xe-ELIPs reduced the extent of damage, decreased cell death, and improved functional outcomes. Of particular significance are large-animal models of TBI, in which Xenon microbubbles were associated with reductions in vasogenic edema, perivascular inflammation, and BBB damage.

Extracranial data remain less developed; the most informative is a renal ischemia–reperfusion model, in which platelet-mimetic microbubbles provided fixation to the damaged endothelium, ultrasound-induced Xenon release, and a reduction in the functional and morphological signs of acute kidney injury. The cardioprotective evidence remains more limited and should currently be interpreted as a preliminary, hypothesis-generating signal rather than as established evidence of extracranial efficacy.

At present, the available evidence should be considered as preclinical proof-of-concept rather than as evidence of translational readiness.

Therefore, potential targets for localized Xenon delivery should be considered in the context of established clinical scenarios in which Xenon has demonstrated cytoprotective efficacy. These include post-resuscitation brain injury, acute cerebrovascular injury, TBI, and selected types of ischemia–reperfusion organ injury. In these settings, intravenous lipid or microbubble formulations could theoretically modify the delivery profile by reducing the absolute amount of gas required, decreasing dependence on prolonged inhalation, and shifting the balance from systemic exposure toward more localized tissue delivery.

However, the expected gas-saving effect has not yet been quantitatively demonstrated. The available studies report Xenon loading, carrier volume, or therapeutic effects, but they do not provide direct comparisons with inhaled Xenon in terms of total Xenon consumption, treatment cost, or pharmacoeconomic efficiency. Therefore, reduced Xenon consumption and economic benefit should currently be regarded as plausible translational advantages that require dedicated validation rather than established outcomes.

Importantly, no direct head-to-head studies have compared inhaled Xenon with carrier-delivered Xenon within the same experimental protocol. Therefore, the potential advantages of liposomal, nanobubble-, or microbubble-mediated Xenon delivery should be interpreted as preclinical feasibility and proof-of-concept evidence rather than as demonstrated superiority over conventional inhalational administration. Experimental models have identified conditions in which targeted Xenon delivery can produce reproducible biological effects, and several potential mechanistic targets have been outlined, including ischemia–reperfusion injury, apoptosis, microcirculatory dysfunction, vascular-barrier damage, and endothelial reactivity. However, further development requires clarification of dose–response relationships, pharmacokinetics, biodistribution, ultrasound release regimens, repeat-dose safety, immunogenicity, and validation in adequately powered large-animal studies. Only after these steps can targeted Xenon delivery be discussed in terms of clinical translation rather than preclinical feasibility.

## Figures and Tables

**Table 1:** Comparative summary of Xenon-containing liposomes, nanobubbles, and microbubbles for targeted cytoprotective delivery.

Platform	Carrier/ Formulation	Xe Loading/ Dose Unit	Trigger/ Localization	Evidence Base	Directly Observed Effects	Main Gaps
Xe-ELIP	Echogenic liposomes; lipid composition varies across studies (e.g., DPPC/DOPC/cholesterol or DPPC/Egg-PC/PEG2000-PE/DPPG/ cholesterol)	Typically ~15–23.38 μL Xe/mg lipid; Miao reports ~20 μL Xe/mg lipid. Doses include 3.5–20 mg/kg or 100–600 μL suspension depending on model	Ultrasound over carotid/internal carotid region; commonly 1 MHz, 0.18 MPa in early stroke studies; 1 MHz, 0.5 W/cm^2^, 5 min in eMCAO/rtPA study	Rat transient MCAO/reperfusion; prolonged 6 h MCAO with repeated dosing; SAH; embolic MCAO + rtPA	Reduced infarct/hemorrhage volume, TUNEL-positive cells, MMP9 signal with rtPA, and improved behavioral outcomes	Best biological evidence base. Main gaps: no direct tissue Xe PK, no head-to-head inhalation comparison, repeat-dose safety and immunogenicity incomplete
Xe-NB	Lipid nanobubbles; DSPE-PEG2K/DSPC; mean diameter about 225 ± 11 nm; particle concentration about 1.95 × 10^9^/mL in the cited study	73 ± 2 μL Xe/mL suspension; 200 μL intravenously in mouse MCAO/reperfusion model	Used for image-guided accumulation/ultrasound visualization; Image-guided accumulation and ultrasound visualization; available parameters are reported according to the cited study.	Mouse 1 h MCAO followed by reperfusion	Accumulation in ischemic region; CBF recovery to ~88 ± 2% of normal vs. ~75–77% in controls; reduced infarct/apoptosis and improved neurological status	Promising microcirculatory/imaging platform. Conversion to μL/mg lipid not possible without lipid concentration; evidence limited to a narrow model set
Xe-MB	Lipid-shell microbubbles; in TBI studies DBPC + DSPE-PEG5000; control gas often perfluorobutane	TBI suspension reported as 4.5 μmol/mL; engineering studies report ~113 μL Xe/mg lipid	Carotid-level ultrasound triggering in piglet TBI studies; a portable ultrasound probe was used for ultrasound-mediated Xe-MB disruption, with available acoustic parameters summarized in Table S1.	Pilot and expanded piglet controlled cortical impact TBI models; engineering characterization studies	Reduced edema/lesion expansion, reactive vascular changes, perivascular inflammation trend/signals, fibrinogen extravasation; in vitro ZO-1 preservation	Large-animal translational bridge. Main gaps: small *n*, limited functional outcomes, cavitation safety, persistence and repeated exposure
Xe-OFP-MB	Lipid-shell microbubbles with Xenon + octafluoropropane (OFP), usually 90:10 gas mixture; DSPC/PEG-lipid shell in Shekhar platform	Reported around 145–151 μL Xe/mg lipid in the cited engineering study	Engineering release under 6 MHz imaging and 220 kHz pulsed ultrasound; OFP improves stability/contrast under undersaturated conditions	Engineering characterization; no definitive therapeutic in vivo neuroprotection model in the current manuscript corpus	Higher loading/stability/contrast than Xe-MB; ultrasound-triggered release demonstrated	Useful platform evidence, not direct therapeutic efficacy evidence. Avoid over-weighting it in biological conclusions
Xe-Pla-MB	Hybrid platelet membrane-mimicking microbubbles; platelet membrane promotes adhesion to injured endothelium; gas mixture includes Xe + C3F8/OFP-like stabilizing component	High specific loading reported (~136.9 μL Xe/mg phospholipid in the report), but exact Xe amount per 400 μL therapeutic dose cannot be calculated without lipid mass in working suspension	Ultrasound destruction over injured kidney for 10 min in renal IR model	Rat renal ischemia–reperfusion injury	Improved creatinine/urea/pathology/fibrosis; decreased senescence markers p53/p16/β-gal; targeting to damaged endothelium	Strong targeting concept, but safety questions are central: thrombogenicity, off-target adhesion, immune clearance, inflamed microvasculature

Abbreviations: Xe, xenon; Xe-ELIP, xenon-containing echogenic liposomes; Xe-NB, xenon-containing nanobubbles; Xe-MB, xenon-loaded microbubbles; Xe-OFP-MB, xenon–octafluoropropane co-loaded microbubbles; Xe-Pla-MB, xenon-loaded platelet membrane-mimicking microbubbles; OFP, octafluoropropane; MCAO, middle cerebral artery occlusion; SAH, subarachnoid hemorrhage; rtPA, recombinant tissue plasminogen activator; CBF, cerebral blood flow; PK, pharmacokinetics; TUNEL, terminal deoxynucleotidyl transferase dUTP nick-end labeling; MMP9, matrix metalloproteinase 9.

**Table 2:** Evidence-based interpretation of directly measured effects, supported mechanisms, and limitations of Xenon-containing carrier platforms.

Carrier/Model	Directly Measured in Cited Studies	Mechanism Supported	Not Directly Demonstrated	Safe Wording
Xe-ELIP; transient MCAO/reperfusion	Infarct volume, behavioral tests, TUNEL; reported BDNF/Akt/MAPK changes in the source literature	Ischemia–reperfusion neuroprotection; anti-apoptotic/survival-signaling support in this model	Direct tissue Xenon exposure; universal microcirculatory mechanism; permanent MCAO protection	“Xe-ELIP reduced infarct volume and apoptosis-related readouts in transient MCAO/reperfusion models”
Xe-ELIP; embolic MCAO + rtPA	Clot lysis compatibility, CBF change, infarct size, TUNEL, MMP9, hemorrhage frequency	Compatibility with thrombolysis; reduced neuronal death and MMP9/barrier-associated hemorrhagic risk	Not an enhancer of thrombolysis; not equivalent to transient MCAO; no direct inhaled comparison	“In embolic MCAO, Xe-liposomes did not impair rtPA activity and reduced infarct/TUNEL/MMP9-associated injury”
Xe-ELIP; SAH	Hemorrhage volume, TUNEL-positive cells, early brain injury markers	Early brain injury/apoptosis-related protection after SAH	Not an MCAO model; not proof of ischemic–reperfusion mechanism	“SAH evidence supports early brain injury reduction after Xe-ELIP, but should be discussed separately from MCAO”
Xe-NB; mouse MCAO/reperfusion	Carrier accumulation/imaging, CBF restoration, infarct volume, apoptosis markers, neurological score	Microcirculatory restoration plus neuroprotection in a mouse reperfusion setting	Not liposome-equivalent; no conversion to μL/mg lipid without lipid mass	“Xe-NB data suggest image-guided delivery and microcirculatory recovery in mouse MCAO/reperfusion”
Xe-MB; piglet TBI	MRI FA/RD/MD/AD, perivascular inflammation, reactive vascular changes, GFAP, fibrinogen extravasation; Iba1 not significant	Vascular-barrier protection: edema, BBB leakage/fibrinogen, endothelial/perivascular changes; astrocytic signal is region-dependent	Universal anti-inflammatory effect; microglial suppression; axonal diffusion benefit	“The piglet TBI data mainly support vascular-barrier effects, not broad anti-inflammatory effects”
Xe-Pla-MB; renal IR	Creatinine, urea, pathology score, NGAL/KIM-1, fibrosis, p53/p16/β-gal senescence markers, endothelial-targeting imaging	Renal endothelial-targeted protection and senescence-marker reduction after kidney IR	Neurovascular mechanism; brain targeting; generalizable anti-senescence across organs	“Renal Xe-Pla-MB data should be kept as organ-specific evidence and not generalized to CNS mechanisms”
Xe-ELIP; myocardial infarction abstract	Reported LV end-systolic volume, cardiac output, infarct volume, but abstract-only and incomplete numerical dataset	Hypothesis-generating cardiac signal only	Established extracranial efficacy; full peer-reviewed cardioprotection evidence	“Cardiac Xe-ELIP evidence is preliminary and should not be weighted equally with full experimental studies”

Abbreviations: BBB, blood–brain barrier; GFAP, glial fibrillary acidic protein; Iba1, ionized calcium-binding adapter molecule 1.

**Table 3:** Therapeutic time window, dose–response, and ultrasound-triggered release effects of Xe-ELIPs in transient MCAO.

Experimental Block	Treatment Group	Xe-ELIP Dose/Injected Volume	Treatment Timing	Ultrasound Exposure	Normalized Infarct Volume, %	Statistical Comparison/Functional Outcome
Therapeutic time window	Untreated MCAO control	—	—	Not applied	16 ± 1.8	Reference group; severe neurological deficit
Therapeutic time window	Xe-ELIP	7 mg/kg; 200 µL	2 h after stroke onset	1 MHz, 0.18 MPa	2.9 ± 0.7	*p* < 0.001 vs. control; improvement in limb placement, beam walking, and grid walking tests from day 1
Therapeutic time window	Xe-ELIP	7 mg/kg; 200 µL	3 h after stroke onset	1 MHz, 0.18 MPa	5.6 ± 1.2	*p* < 0.001 vs. control; improvement in behavioral tests, particularly by day 3
Therapeutic time window	Xe-ELIP	7 mg/kg; 200 µL	5 h after stroke onset	1 MHz, 0.18 MPa	8.5 ± 1.3	*p* = 0.004 vs. control; moderate behavioral improvement, weaker than with earlier administration
Dose–response	Xe-ELIP	3.5 mg/kg; 100 µL	3 h after stroke onset	1 MHz, 0.18 MPa	12 ± 3.0	*p* > 0.05 vs. corresponding control; no significant behavioral improvement
Dose–response	Xe-ELIP	7 mg/kg; 200 µL	3 h after stroke onset	1 MHz, 0.18 MPa	5.6 ± 1.2	*p* < 0.001 vs. corresponding control; significant behavioral improvement
Dose–response	Xe-ELIP	14 mg/kg; 400 µL	3 h after stroke onset	1 MHz, 0.18 MPa	3.3 ± 0.7	*p* < 0.001 vs. corresponding control; strongest reduction in infarct volume
Ultrasound-triggered release	Untreated MCAO control	—	—	Not applied	21 ± 2.5	Reference group for ultrasound comparison
Ultrasound-triggered release	Xe-ELIP without ultrasound	7 mg/kg; 200 µL	3 h after stroke onset	Not applied	6.1 ± 0.5	*p* < 0.001 vs. control; Xenon-loaded carrier retained neuroprotective activity without triggered release
Ultrasound-triggered release	Xe-ELIP + ultrasound	7 mg/kg; 200 µL	3 h after stroke onset	1 MHz, 0.18 MPa	2.9 ± 0.7	*p* < 0.001 vs. control; ultrasound-triggered release further enhanced neuroprotection

Note. All data are from Peng et al. [24]. The summarized experiments were performed in rats subjected to 2 h transient middle cerebral artery occlusion followed by reperfusion; therefore, this table refers only to transient MCAO and should not be interpreted as evidence from permanent MCAO. Xe-ELIPs were administered intravenously. Treatment timing is counted from stroke onset, not from reperfusion; because MCAO lasted 2 h, the 2 h treatment point corresponds to treatment at the onset of reperfusion, whereas the 3 h and 5 h treatment points correspond to 1 h and 3 h after reperfusion, respectively. In rows with ultrasound exposure, ultrasound was applied over the carotid artery during Xe-ELIP administration to trigger Xenon release. Infarct volume was assessed by TTC staining on day 3 and is presented as normalized infarct volume, %, with mean ± SEM. Behavioral outcomes were assessed on days 1–3 using limb placement, beam walking, and grid walking tests. The untreated control values are block-specific and should not be pooled: 16 ± 1.8% refers to the therapeutic time-window block, whereas 21 ± 2.5% refers to the ultrasound-triggered release block. Dose–response *p* values are reported versus the corresponding untreated MCAO control in the original dose–response experiment. Group size was *n* = 8 per group in the original experimental blocks; *n* is not repeated in the table because all rows derive from the same source and model. The ultrasound-triggered release comparison corresponds to 7 mg/kg Xe-ELIP (200 µL), administered 3 h after stroke onset, not 14 mg/kg Xe-ELIP (200 µL). MCAO, middle cerebral artery occlusion; Xe-ELIP, xenon-containing echogenic liposome; TTC, 2,3,5-triphenyltetrazolium chloride.

**Table 4:** Behavioral test results.

Test and Indicator	Control	Sham Surgery	MCAO Group	Xe-ELIP	Significance
Elevated Plus Maze: Time in the Open Arms, %	≈3.3	≈5.0	≈0.8–1.0	≈9.0	The overall effect: F(4.37) = 7.738; *p* = 0.00011
Elevated Plus Maze: Open Arm Entrances, %	≈10	≈13	≈2	≈14%	The overall effect: F(4.37) = 3.295; *p* = 0.02092
Elevated Plus Maze: Entrances to Closed Arms, %	≈90	≈86–87	≈97–98	≈85–86	*p* > 0.053
Forced Swim Test: Active Swim Time, sec	≈78	≈99–100	≈51–52	≈105	*p* < 0.00124

Note: Results are based on the graphical data from the original article [43] and are therefore marked as approximate.

**Table 5:** The schedule of the experiment at the in vivo stage.

120 min		240 min	
Time	Action	Time	Action
0 min	Embolic occlusion of the MCA	0 min	Embolic occlusion of the MCA
110 min	Xe-ELIP injection in the relevant group	110 min	Xe-ELIP injection in the relevant group
120 min	Start of thrombolysis	4 h	Start of thrombolysis
120–180 min	Alteplase infusion within 60 min	4–5 h	Alteplase infusion within 60 min
1–3 days	Behavioral tests	1–3 days	Behavioral tests
3rd day	TTC infarction score	3rd day	TTC, TUNEL, hemorrhagic component, MMP9

**Table 6:** Preclinical evidence, mechanistic support, and interpretation of Xenon-containing carriers in cerebrovascular injury models.

Study/Carrier	Injury/Reperfusion Phenotype	Experimental Emphasis	Main Positive Signal	Mechanistic Signal Supported by the Study	Interpretation for This Review
Britton et al., Xe-ELIP	2 h transient MCAO followed by reperfusion	First in vivo proof-of-concept for ultrasound-triggered Xe-ELIP delivery	Reduced normalized infarct volume; effect enhanced by ultrasound	Local carrier release; reduction in ischemic tissue injury	Demonstrates feasibility of intravenous Xe-ELIPs with ultrasound-triggered release in transient ischemia–reperfusion
Peng et al., Xe-ELIP	2 h transient MCAO followed by reperfusion	Therapeutic window and dose–response	Protection preserved up to 5 h; higher Xe-ELIP dose produced stronger reduction in infarct volume	Reduced infarct volume and behavioral improvement; molecular survival/apoptosis signals reported	Defines dose/window relationships within transient MCAO, not permanent MCAO
Dandekar et al., repeated Xe-ELIP	Prolonged 6 h transient MCAO followed by reperfusion	Repeated dosing and long-term behavioral outcome	Improved sensorimotor recovery and anxiety/depressive-like behavioral endpoints	Long-term functional recovery; cytokine/microbiota signals exploratory	Extends Xe-ELIP evidence to a more severe prolonged ischemia–reperfusion paradigm
Peng et al., Xe-liposomes + rtPA	Embolic MCAO with pharmacological reperfusion	Compatibility with thrombolysis and late rtPA-associated injury	Combination with rtPA reduced infarct volume; late treatment reduced hemorrhagic complications, TUNEL-positive cells, and MMP9 signal	Apoptosis and vascular-barrier protection in the context of thrombolysis	Supports adjunctive use with reperfusion therapy; mechanistically distinct from filament transient MCAO
Miao et al., Xe-ELIP	Subarachnoid hemorrhage by endovascular perforation	Early brain injury after hemorrhagic cerebrovascular damage	Reduced hemorrhage volume and TUNEL-positive cells; empty carriers and Xe-saturated solution were ineffective	Reduced early cell death after SAH	Should be discussed as hemorrhagic brain injury, not as MCAO/stroke ischemia model
Jin et al., Xe-NB	1 h transient MCAO followed by reperfusion in mice	Nanobubble accumulation, ultrasound imaging, microcirculatory recovery	Nanobubbles accumulated in ischemic tissue, improved microcirculatory recovery, reduced infarct/apoptosis, improved neurological status	Microcirculatory restoration and neuroprotection	Represents a distinct nanobubble ischemia–reperfusion platform, not directly comparable with Xe-ELIP dose–response studies

Note. MCAO, middle cerebral artery occlusion; SAH, subarachnoid hemorrhage; Xe-ELIP, xenon-containing echogenic liposomes; Xe-NB, xenon-containing nanobubbles. The studies summarized in this table are grouped by injury/reperfusion phenotype rather than by chronological order to clarify model heterogeneity. The narrative order in Section 4.1 is retained to reflect the historical development of the field. Permanent MCAO models were not identified among the carrier-delivered Xenon studies analyzed in this review; therefore, the available evidence should be interpreted primarily in the context of transient ischemia–reperfusion, prolonged transient ischemia with reperfusion, embolic stroke with pharmacological reperfusion, and hemorrhagic brain injury.

**Table 7:** The key stages of the research.

Stage	Purpose	Scheme	Evaluation Period	Primary Endpoint
Xenon before and after injury	To check the basic protection with the most optimal circuit	75% Xenon/25% O_2_ 2 h before injury and 2 h after TBI	24 h	Neurological outcome, contusion volume
Xenon only after injury	To test a clinically more realistic option for early initiation of therapy	75% Xenon/25% O_2_, onset 15 min after TBI, duration 3 h	24 h	Neurological outcome, contusion volume
Therapeutic window	To determine the effectiveness of delayed onset	75% Xenon/25% O_2_, onset 15 min, 1 h, 3 h or 6 h after TBI, duration 3 h	24 h	Neurological outcome, contusion volume
Application of different concentrations	Assessment of dose–response effect	30%, 50%, or 75% Xenon with 25% O_2_, onset at 15 min, duration 3 h	24 h	Contusion volume, neurological outcome
Physiological assessment	To eliminate impact on system performance	75% Xenon/25% O_2_ vs. control gas	During exposure	Blood pressure, heart rate, temperature
Dynamics up to 5 days	To check the preservation of the early functional effect	75% Xenon/25% O_2_, onset 15 min, duration 3 h	Days 1–5	Neurological outcome
Long-term motor effects	Assessment of the durability of effects	75% Xenon/25% O_2_, onset 15 min, duration 3 h	1 month	Rotarod, automated gait analysis

**Table 8:** The results of the use of Xenon.

Indicator	Control Microbubbles with Perfluorobutane	Xe-MBa	*p*
Change in edema volume according to MRI from 1 to 5 days	An increase of approximately 160%	Around 0%/slight decrease	≤0.03
Changes in the nucleus and hemorrhagic component according to MRI	Increase of about 75%	A reduction of approximately 40%	The difference is statistically insignificant
Change in the total volume of damage according to MRI	An increase of approximately 145%	Around −10%	≤0.01
Reactive vascular changes and endothelial proliferation	≈0.85 points	≈0.15 points	0.002
Perivascular inflammation	≈1.2 points	≈0.15 points	Lower in the Xenon group but without statistical significance

Note: Perivascular inflammation was graded from 0 to 3, where 0 is absent/single cells and 3 is an inflammatory cuff of more than one layer. Reactive vascular changes were graded from 0 to 2, where 0 is no changes, 1 is reactive endothelial cells, and 2 is endothelial proliferation of more than one cell layer.

**Table 9:** In vivo results outcomes of Xe-MBs versus control microbubbles in a porcine traumatic brain injury model.

Evaluation Domain	Direct Endpoint/Method	Endpoint Timing	*n* Used for This Endpoint	Direct Factual Result/Statistical Comparison
White matter diffusion	Fractional anisotropy, diffusion tensor MRI	Day 1	CtMB *n* = 5; Xe-MB *n* = 8	Fractional anisotropy was higher in the Xe-MB group than in the CtMB group in both corona radiata, both cerebral peduncles, and in the genu and splenium of the corpus callosum; *p* < 0.05 for the indicated regions
White matter diffusion	Fractional anisotropy, diffusion tensor MRI	Day 5	CtMB *n* = 5; Xe-MB *n* = 8	Most day 1 differences were no longer present; higher fractional anisotropy persisted only in a small region of the right cerebral peduncle; *p* < 0.05
Vasogenic edema-related diffusion	Radial diffusivity, diffusion tensor MRI	Day 1	CtMB *n* = 5; Xe-MB *n* = 8	Radial diffusivity was higher in the CtMB group than in the Xe-MB group in both corona radiata, the genu and splenium of the corpus callosum, and the contralateral cerebral peduncle; *p* < 0.05 for the indicated regions
General tissue diffusion	Mean diffusivity, diffusion tensor MRI	Day 1	CtMB *n* = 5; Xe-MB *n* = 8	Mean diffusivity was lower in the Xe-MB group only in the contralateral corona radiata; *p* < 0.05
General tissue diffusion	Mean diffusivity, diffusion tensor MRI	Day 5	CtMB *n* = 5; Xe-MB *n* = 8	No significant differences between groups were detected; *p* > 0.05
Axial diffusion	Axial diffusivity, diffusion tensor MRI	Days 1 and 5	CtMB *n* = 5; Xe-MB *n* = 8	No significant differences between groups were detected at either time point; *p* > 0.05
Perivascular inflammation	H&E staining; semi-quantitative perivascular inflammation score	Day 5	CtMB *n* = 4; Xe-MB *n* = 8	On the injured side, perivascular inflammation was lower in the Xe-MB group than in the CtMB group; *p* < 0.05. On the contralateral side, the difference did not reach statistical significance; *p* = 0.052
Reactive vascular changes	H&E staining; semi-quantitative endothelial reactivity/vascular proliferation score	Day 5	CtMB *n* = 4; Xe-MB *n* = 8	On the injured side, reactive vascular changes were lower in the Xe-MB group than in the CtMB group; *p* < 0.05
Astrocytic reactivity	GFAP immunostaining; percentage of stained area	Day 5	CtMB *n* = 4; Xe-MB *n* = 8	GFAP-positive stained area was lower in the Xe-MB group in the contralateral cortex and ipsilateral white matter; *p* < 0.05. Differences in the ipsilateral cortex and contralateral white matter did not reach statistical significance; *p* = 0.08 and *p* = 0.06, respectively
Microglial reactivity	Iba1 immunostaining; percentage of stained area	Day 5	CtMB *n* = 4; Xe-MB *n* = 8	No significant differences between groups were detected; *p* > 0.05
Blood–brain barrier disruption	Fibrinogen extravasation immunostaining	Day 5	CtMB *n* = 4; Xe-MB *n* = 8	Fibrinogen extravasation was lower in the Xe-MB group in ipsilateral white matter; *p* = 0.01. In the perifocal cortex, the difference did not reach statistical significance; *p* = 0.17. Contralateral staining was minimal, without significant group differences

Note. All data are from Shin et al. [42]. The in vivo experiment used a controlled cortical impact model of traumatic brain injury in piglets. The treatment comparison was Xenon-containing microbubbles (Xe-MBs) versus control perfluorobutane-containing microbubbles (CtMBs). Xe-MBs or CtMBs were administered intravenously at 1, 3, and 24 h after TBI. The reported Xenon dose was approximately 1.5 mL Xe per animal in total, divided into three administration cycles of approximately 0.5 mL Xe each; each cycle used approximately 4 mL of microbubble infusate. Ultrasound was applied at the carotid artery level during microbubble administration to trigger intravascular Xenon release before cerebral entry; detailed acoustic parameters such as frequency, acoustic pressure, and mechanical index were not specified in the available manuscript text. DTI was performed on days 1 and 5 after injury; histological and immunohistochemical endpoints were assessed after euthanasia on day 5. CtMB, control perfluorobutane-containing microbubble; Xe-MB, xenon-containing microbubble; TBI, traumatic brain injury; DTI, diffusion tensor imaging; MRI, magnetic resonance imaging; H&E, hematoxylin and eosin; GFAP, glial fibrillary acidic protein; Iba1, ionized calcium-binding adaptor molecule 1.

## Data Availability

No new data were created or analyzed in this study. Data sharing is not applicable to this article.

## Supplementary Materials

The following supporting information can be downloaded at https://www.mdpi.com/article/10.3390/pharmaceutics18070855/s1, Table S1: Reported ultrasound-triggering parameters used for Xenon-loaded carrier systems.
