# Supplementary material for: Xenon Encapsulation in Liposomes, Nanobubbles, and Microbubbles: Delivery Strategies, Preclinical Evidence, and Translational Barriers
Source: Pharmaceutics. 2026 Jul 14;18(7):855. doi: 10.3390/pharmaceutics18070855 (PMC13414817; doi:10.3390/pharmaceutics18070855)
Supplement: Supplementary file 1 [file pharmaceutics-18-00855-s001.zip › pharmaceutics-4431128-supplementary.pdf]

Xenon Encapsulation in Liposomes, Nanobubbles, and Microbubbles: Delivery Strategies, Preclinical Evidence, and Translational Barriers

Rostislav A. Cherpakov[1,*,†], Vera S. Shashkovskaya[1,†], Oleg A. Grebenchikov[1, ‡] and Viktoria Sergunova[1,*, ‡]

Supplementary Table S1. Reported ultrasound-triggering parameters used for xenon-loaded carrier systems

Study / source	Carrier exposed to ultrasound	Experimental setting	Purpose of ultrasound exposure	Exposure site / target	Frequency	Acoustic pressure / intensity / MI	Mode / duty cycle	Irradiation duration / timing	Reporting comment
Britton et al., 2010 [1]	Xe-ELIP	Rat 2-h transient MCAO followed by reperfusion	Ultrasound-triggered xenon release from circulating Xe-ELIP	Internal carotid artery region	1 MHz	0.18 MPa peak-to-peak pressure amplitude	Continuous wave	During Xe-ELIP administration	Low-amplitude continuous ultrasound increased xenon release from Xe-ELIP compared with no ultrasound.
Peng et al., 2013 [2]	Xe-ELIP	Rat 2-h transient MCAO followed by reperfusion; therapeutic-window and dose-response experiments	Ultrasound-triggered xenon release	Common carotid artery region	1 MHz	0.18 MPa peak-to-peak pressure amplitude	Continuous wave	During Xe-ELIP administration	Same low-amplitude Xe-ELIP ultrasound-triggering strategy as in the early stroke protocol.

Dandekar et al., 2022 [3]	Repeated Xe-ELIP / xenon-loaded liposomes	Rat prolonged 6-h MCAO followed by reperfusion; repeated dosing	Ultrasound-triggered release during serial carrier administration	Internal carotid artery region	N.R.	N.R.	Low-intensity ultrasound; exact parameters not extracted	During each administration cycle	Do not infer exact frequency, pressure, or duration unless verified directly from the original full text.
Peng et al., 2023 [4]	Xenon-loaded liposomes + rtPA	Rat embolic MCAO with early or delayed thrombolysis	Ultrasound-triggered xenon release in combination with rtPA	Common carotid artery region	1 MHz	0.5 W/cm²	Continuous wave; 100% duty cycle	5 min during Xe-liposome administration; Xe-liposomes were administered 5 min before rtPA	This study should be cited separately from the SAH Xe-ELIP paper during the reference audit.
Miao et al., 2018 [5]	Xe-ELIP	Rat subarachnoid hemorrhage	Ultrasound-triggered xenon release after intravenous Xe-ELIP administration	Left internal carotid artery region; probe 5 mm above the artery	1 MHz	0.18 MPa peak-to-peak pressure amplitude	Continuous wave	During 15-min liposomal infusion	Xe-ELIP, empty ELIP, or Xe-saturated solution were infused over 15 min; ultrasound was applied during infusion.
Raymond et al., 2016 [6]	ELIP, mechanistic acoustic study	In vitro single-ELIP acoustic imaging	Observation of ELIP gas loss and destruction mechanisms	In vitro optical / high-speed imaging setup	6 MHz	Pressure varied; exact values should be checked in full text before tabulation	Pulsed ultrasound bursts	During ultra-high-speed recordings	Mechanistic ELIP study; supports discussion of gas loss, fragmentation, and pressure-dependent behavior, but is not a therapeutic xenon-delivery experiment.

Shekhar et al., 2019 [7]	Xe-MB and Xe-OFP-MB	Engineering characterization; no therapeutic injury model	Microbubble imaging and ultrasound-triggered gas release	In vitro acoustic characterization setting	6 MHz; 220 kHz	Primary accessible abstract: N.R.; secondary review reports MI 0.8 for 6 MHz Doppler and MI 0.47 for 220 kHz pulsed ultrasound	6-MHz duplex Doppler; 220-kHz pulsed ultrasound	Secondary review reports 10 s	Primary source confirms 6 MHz and 220 kHz release testing. MI and 10-s duration should be treated as secondary-extracted values unless verified directly from the full article.
Jin et al., 2021 [8]	Xe-NB	Mouse 1-h MCAO followed by reperfusion	Ultrasound visualization of xenon nanobubble accumulation in ischemic tissue	Ischemic brain region	N.R.	N.R.	Ultrasound imaging / contrast visualization	Imaging after intravenous injection	This study supports image-guided accumulation and microcirculatory recovery, but should not be used to define a therapeutic ultrasound-release safety range.
Hwang et al., 2022 [9]	Xe-MB	Porcine controlled cortical impact TBI	Ultrasound-triggered xenon release into the cerebral circulation	Carotid artery region, longitudinal axis	5–10 MHz	MI = 1.0	Hand-held Lumify Philips ultrasound probe; 18–20 frames/s	During each 8-min microbubble infusion at 1, 3, and 24 h after TBI	Exact large-animal Xe-MB ultrasound-guided release parameters are available and

									should be reported.
Shin et al., 2023 [10]	Xe-MB	Porcine controlled cortical impact TBI	Ultrasound-triggered xenon release before cerebral entry	Carotid artery region	N.R.	N.R.	Carotid-level ultrasound triggering based on the prior Xe-MB workflow	During three administration cycles at 1, 3, and 24 h after TBI	The study follows the Hwang-style large-animal workflow, but exact acoustic parameters should not be repeated as independently reported unless confirmed in the full text.
Yang et al., 2023 [11]	Xe-Pla-MB	Rat renal ischemia-reperfusion injury	Destructive ultrasound exposure to release xenon from platelet membrane-mimicking microbubbles	Injured kidney	N.R.	N.R.	Destructive ultrasound exposure using a clinical ultrasound system	10 min after intravenous Xe-Pla-MB administration	Therapeutic effect depended on the combination of xenon, platelet-mimetic targeting, and ultrasound-triggered carrier destruction; exact frequency and acoustic output should be marked as not reported if not found in the full text.

1. Britton, G.L.; Kim, H.; Kee, P.H.; Aronowski, J.; Holland, C.K.; McPherson, D.D.; Huang, S.-L. In Vivo Therapeutic Gas Delivery for Neuroprotection With Echogenic Liposomes. *Circulation* **2010**, *122*, 1578–1587, doi:10.1161/CIRCULATIONAHA.109.879338.
2. Peng, T.; Britton, G.L.; Kim, H.; Cattano, D.; Aronowski, J.; Grotta, J.; McPherson, D.D.; Huang, S. Therapeutic Time Window and Dose Dependence of Xenon Delivered via Echogenic Liposomes for Neuroprotection in Stroke. *CNS Neurosci Ther* **2013**, *19*, 773–784, doi:10.1111/cns.12159.
3. Dandekar, M.P.; Yin, X.; Peng, T.; Devaraj, S.; Morales, R.; McPherson, D.D.; Huang, S. Repetitive Xenon Treatment Improves Post-Stroke Sensorimotor and Neuropsychiatric Dysfunction. *Journal of Affective Disorders* **2022**, *301*, 315–330, doi:10.1016/j.jad.2022.01.025.
4. Peng, T.; Booher, K.; Moody, M.R.; Yin, X.; Aronowski, J.; McPherson, D.D.; Savitz, S.I.; Kim, H.; Huang, S.-L. Enhanced Cerebroprotection of Xenon-Loaded Liposomes in Combination with rtPA Thrombolysis for Embolic Ischemic Stroke. *Biomolecules* **2023**, *13*, 1256, doi:10.3390/biom13081256.
5. Miao, Y.-F.; Peng, T.; Moody, M.R.; Klegerman, M.E.; Aronowski, J.; Grotta, J.; McPherson, D.D.; Kim, H.; Huang, S.-L. Delivery of Xenon-Containing Echogenic Liposomes Inhibits Early Brain Injury Following Subarachnoid Hemorrhage. *Sci Rep* **2018**, *8*, 450, doi:10.1038/s41598-017-18914-6.
6. Raymond, J.L.; Luan, Y.; Peng, T.; Huang, S.-L.; McPherson, D.D.; Versluis, M.; de Jong, N.; Holland, C.K. Loss of Gas from Echogenic Liposomes Exposed to Pulsed Ultrasound. *Phys Med Biol* **2016**, *61*, 8321–8339, doi:10.1088/0031-9155/61/23/8321.
7. Shekhar, H.; Palaniappan, A.; Peng, T.; Lafond, M.; Moody, M.R.; Haworth, K.J.; Huang, S.; McPherson, D.D.; Holland, C.K. Characterization and Imaging of Lipid-Shelled Microbubbles for Ultrasound-Triggered Release of Xenon. *Neurotherapeutics* **2019**, *16*, 878–890, doi:10.1007/s13311-019-00733-4.
8. Jin, J.; Li, M.; Li, J.; Li, B.; Duan, L.; Yang, F.; Gu, N. Xenon Nanobubbles for the Image-Guided Preemptive Treatment of Acute Ischemic Stroke via Neuroprotection and Microcirculatory Restoration. *ACS Appl. Mater. Interfaces* **2021**, *13*, 43880–43891, doi:10.1021/acsami.1c06014.
9. Hwang, M.; Chattaraj, R.; Sridharan, A.; Shin, S.S.; Viaene, A.N.; Haddad, S.; Khrichenko, D.; Sehgal, C.; Lee, D.; Kilbaugh, T.J. Can Ultrasound-Guided Xenon Delivery Provide Neuroprotection in Traumatic Brain Injury? *Neurotrauma Reports* **2022**, *3*, neur.2021.0070, doi:10.1089/neur.2021.0070.
10. Shin, S.S.; Chattaraj, R.; Viaene, A.N.; Karmacharya, M.B.; Haddad, S.; Degani, R.; Sridharan, A.; Sehgal, C.; Lee, D.; Kilbaugh, T.J.; et al. Brain Targeted Xenon Protects Cerebral Vasculature After Traumatic Brain Injury. *Journal of Neurotrauma* **2023**, *40*, 1470–1480, doi:10.1089/neu.2022.0468.

11. Yang, J.; Chen, C.; Miao, X.; Wang, T.; Guan, Y.; Zhang, L.; Chen, S.; Zhang, Z.; Xia, Z.; Kang, J.; et al. Injury Site Specific Xenon Delivered by Platelet Membrane-Mimicking Hybrid Microbubbles to Protect Against Acute Kidney Injury via Inhibition of Cellular Senescence. *Adv Healthcare Materials* **2023**, *12*, 2203359, doi:10.1002/adhm.202203359.
